# Celebrating 5 years of *Communications Chemistry*

**DOI:** 10.1038/s42004-023-00832-6

**Published:** 2023-03-06

## Abstract

As *Communications Chemistry* turns five, the Editors reflect on the journal’s values, achievements, and ambitions for the years to come.

When launching a new journal, it’s imperative to think about what values it will embody, and what role it will serve in the community. Five years ago, our vision was for *Communications Chemistry* to provide a home for research of significant interest to focused communities within the chemical sciences, whilst delivering the highest standards of editorial practices and publication ethics to ensure quality in content as well as author experience. To put these goals to action, *Communications Chemistry* retains the traditional *Nature* model of in-house professional editors—PhD-level scientists with research experience who are comprehensively trained in all aspects of manuscript handling—and compliments it with an Editorial Board of active researchers recognized as experts in their field.

Five years later, we are truly proud of what *Communications Chemistry* has become. We have published over 800 open access articles, covering the entire breadth of chemistry, while also touching on adjacent areas such as materials science, nanotechnology and chemical engineering. Every publication has been carefully selected for inclusion within the journal, and we are delighted to see the impact that many of these are having on ongoing research in the chemical sciences. Indeed, it is exciting to see that our content is primarily being cited in highly visible and reputable outlets (Fig. 1).

**Fig. 1:**
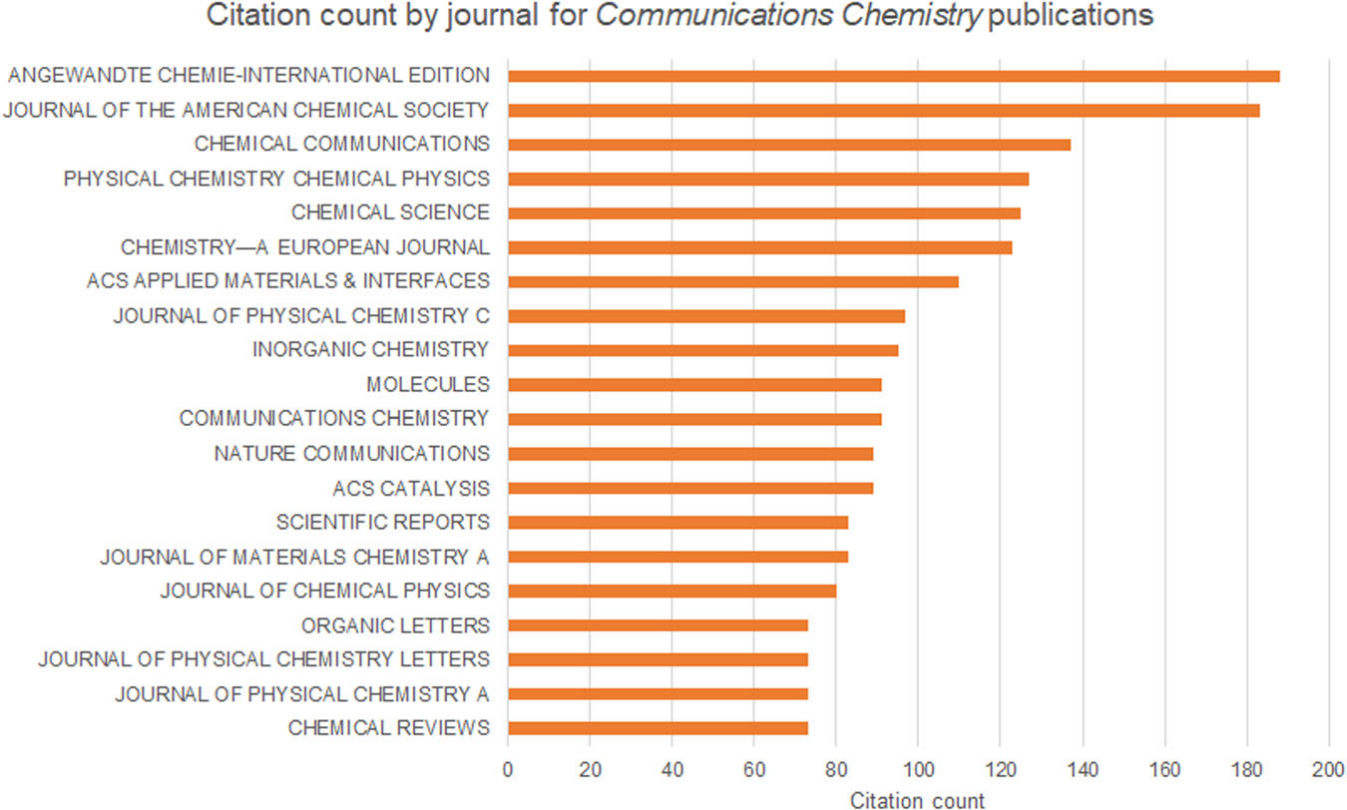
Top 20 journals with highest number of citations to *Communications Chemistry* content. Data retrieved from Web of Science on 31 January 2023, for content published since the journal’s launch on 8 March 2018.

Our wide-ranging article types additionally allow for diverse publications of community voices, through Reviews, Perspectives, Comments, Research Highlights, Q&As and Meeting Reports. Our Comment section in particular has allowed us to cover important topics such as diversity, equity and inclusion (DEI), and education and public outreach. Our Open questions in chemistry series hosts over 30 opinion pieces detailing key fundamental open questions in focused fields of research.

Our journal would be (literally) nothing without our authors and reviewers. We extend a huge thank you to all those who have published with and reviewed for us over the years, in particular those authors who have published with us on numerous occasions, and those reviewers highlighted through our outstanding referees scheme, for going above and beyond what is expected of a reviewer. We also wish to extend an enormous thanks to our Editorial Board Members, and of course to our readers, for supporting the journal from day one.

Today, we are pleased to launch our five year anniversary Collection, featuring a selection of some of the most highly downloaded, cited and talked about content from the last five years. We hope you enjoy reading these papers as much as we enjoyed publishing them.

While we are proud of how far we have come, we still have plenty that we hope to achieve. We hope to build on our previous and existing DEI-oriented initiatives, such as our Women in Chemistry Collection, or our Early Career Researcher travel grants, and would like to invite researchers to put themselves forward to be profiled on their research and lived experiences in a series of Q&A articles that we intend to publish in the journal.

The United Nations’ Sustainable Development Goals are also front and center in our minds. We’re looking forward to seeing our recently launched Guest Edited Collections on Electrocatalytic CO_2_ reduction and Organomediated polymerization come to fruition, and we hope to do more in the coming years to support and surface important contributions that chemical research has to offer to achieve these vital goals.

We also wish to further expand our data and reproducibility policies to strengthen our quality pledge and reflect the needs of the chemistry community. We have already implemented standards for custom computer code, NMR spectra, and electrocatalytic nitrogen fixation to ensure that we support reproducible research and open data in line with community gold standards.

As we continue to grow, we are committed to maintaining our competitive turnaround times, strong editorial guidance, and the excellent quality of our output. We greatly look forward to continuing to work with you all, and to hopefully welcoming many new faces into our (web)pages in the years to come!

